# Energy Consumption Prediction of Injection Molding Process Based on Rolling Learning Informer Model

**DOI:** 10.3390/polym16213097

**Published:** 2024-11-02

**Authors:** Jianfeng Huang, Yi Li, Xinyuan Li, Yucheng Ding, Fenglian Hong, Shitong Peng

**Affiliations:** Department of Mechanical Engineering, Shantou University, Shantou 515063, China; jfhuang@stu.edu.cn (J.H.); 19yli6@stu.edu.cn (Y.L.); 22xyli3@stu.edu.cn (X.L.); 19ycding@stu.edu.cn (Y.D.); 21flhong@alumni.stu.edu.cn (F.H.)

**Keywords:** injection molding process, green manufacturing, data-driven, energy consumption prediction, RL-Informer

## Abstract

Accurate energy consumption prediction in the injection molding process is crucial for optimizing energy efficiency in polymer processing. Traditional parameter optimization methods face challenges in achieving optimal energy prediction due to complex energy transmission. In this study, a data-driven approach based on the Rolling Learning Informer model is proposed to enhance the accuracy and adaptability of energy consumption forecasting. The Informer model addresses the limitations of long-sequence prediction with sparse attention mechanisms, self-attention distillation, and generative decoder techniques. Rolling learning prediction is incorporated to enable continuous updating of the model to reflect new data trends. Experimental results demonstrate that the RL-Informer model achieves a normalized root mean square error of 0.1301, a root mean square error of 0.0758, a mean absolute error of 0.0562, and a coefficient of determination of 0.9831 in energy consumption forecasting, outperforming other counterpart models like Gated Recurrent Unit, Temporal Convolutional Networks, Long Short-Term Memory, and two variants of the pure Informer models without Rolling Learning. It is of great potential for practical engineering applications.

## 1. Introduction

Plastic is one of the most versatile materials in modern life, integral to numerous industries and consumer products, with global production exceeding 400 million metric tons in 2024 [1]. Within the plastic industry, injection molding is a critical process that is not only energy-intensive but also marked by large-scale production and relatively low energy efficiency, often requiring between 4 and 5 kWh of energy per kilogram of plastic produced [2]. The accurate prediction of energy consumption in injection molding enables timely adjustments to operational parameters and process optimization [3], leading to reduced energy consumption and significant improvements in both energy efficiency and production output.

**Figure 1:**
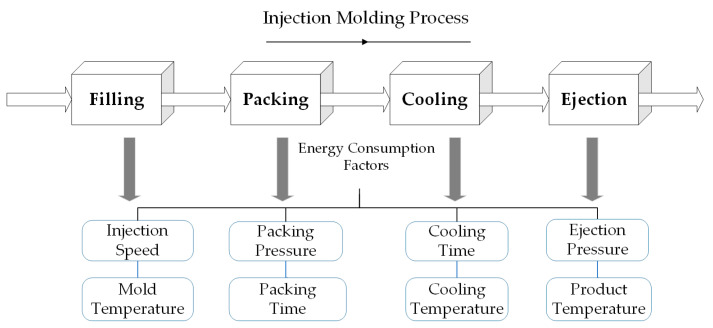
Injection molding steps and energy consumption influencing factors.

The injection molding process is typically divided into four key stages: filling, packing, cooling, and ejection [4]. Research has shown that each of these stages involves multiple factors that significantly affect energy consumption (see Figure 1), such as filling speed and mold temperature during the filling phase [5]. Meekers et al. identified that different factors have varying degrees of impact on energy consumption, with cycle time having the most pronounced effect, followed by nozzle temperature [6].

To date, numerous researchers have investigated the factors influencing energy consumption in injection molding, utilizing statistical models, machine learning techniques, and simulation modeling to predict energy usage in this process. For example, Pacella et al. employed a regression mixed model based on time series clustering to classify energy consumption patterns and construct virtual energy profiles for predictive purposes [7]. Takasaki et al. incorporated actual operational parameters into a simulated injection molding framework, enabling forecasts under various conditions [8]. However, due to the complex, nonlinear relationships between parameters like temperature, pressure, and cooling time [9], traditional models often fall short in accuracy, while high-fidelity simulations require substantial computational resources and data. This highlights an urgent need for more efficient analytical methods to develop predictive energy models for injection molding. To address these challenges, researchers have increasingly employed machine learning models to uncover latent relationships and predict energy consumption. For example, Bahij et al. evaluated the performance of machine learning techniques, such as artificial neural networks (ANNs) and support vector regression (SVR), in comparison with traditional methods like rule-based systems in industrial settings. Their results demonstrated that machine learning approaches significantly outperform traditional methods [10], both in terms of accuracy and efficiency, particularly when modeling complex energy consumption patterns and managing the nonlinear dynamics inherent to industrial energy use.

In the injection molding process, variables that influence energy consumption usually show complex correlations that machine learning models can effectively capture [11]. For example, Willenbacher et al. utilized the random forest algorithm to identify key factors affecting energy consumption in the production of plastic parts, enabling accurate predictions of energy usage and the determination of optimal machine settings to minimize energy consumption [12]. Similarly, Wu et al. employed an active machine learning approach to optimize the process parameters of injection molding machines, achieving a multi-objective optimization that balances energy consumption and part quality. Their research demonstrated that the trained energy consumption prediction model possesses robust predictive capabilities [13].

However, industrial data are often complex, nonlinear, and multidimensional challenging machine learning models like random forests, which require large datasets and perform poorly with noisy data or time series characteristics, thus limiting prediction accuracy. Neural networks, however, can effectively model nonlinear relationships and long-term dependencies, adapting to data variations to predict energy consumption accurately across different production settings. For instance, Nazir et al. demonstrated that the TFT achieved superior predictive performance with a symmetric mean absolute percentage error of 26.46%, outperforming LSTM (29.78%), interpretable LSTM (31.10%), and TCN (36.42%) [14].

The self-attention mechanism inherent in the Transformer model enables it to focus on relevant segments of the input sequence, effectively managing long-distance dependencies in time series data, which is particularly advantageous for energy prediction over extended periods [15]. However, traditional Transformer models exhibit high computational complexity and low inference efficiency [16] when addressing multivariate long-sequence predictions. To address these efficiency challenges, the Informer model incorporates advanced techniques [17], including a probabilistic self-attention mechanism, self-attention distillation, and a generative decoder. These enhancements result in significant improvements in both the accuracy and inference efficiency of multivariate long-sequence predictions, thereby increasing its practical applicability in energy forecasting.

To summarize, research issues that motivate the presents study are (1) Traditional machine learning methods can hardly capture the complex temporal features associated with energy consumption predictions in the injection molding process. (2) Simulation modeling requires substantial amounts of practical data, making it challenging to predict dynamic changes in energy consumption. (3) Transformer models are good at capturing temporal relationships but suffer from computation complexity for long sequences, which makes them difficult for practical deployment in the injection molding process. To bridge the research gap and enable more accurate energy prediction in the injection molding process, we propose the following contributions: We first conducted injection molding experiments to identify multiple groups of variables that may influence energy consumption. We then performed a correlation analysis on the experimental variables, selecting those with high correlations to energy consumption in order to reduce model complexity. Finally, we proposed an improved RL-Informer model to predict energy consumption during the injection molding process, which excels at capturing the intricate temporal dependencies within the data while maintaining a low computational complexity. A comparative analysis was performed between the improved RL-Informer model and other models, including LSTM and GRU, highlighting the advantages of the enhanced Informer model. Through this study, we aim to optimize production strategies, facilitate real-time monitoring, and enable predictive maintenance, ultimately contributing to more efficient and environmentally sustainable manufacturing practices.

The structure of this study is organized as follows: Section 2 describes the data collection and preprocessing methods employed in this research. Section 3 elaborates on the sparse attention mechanism, self-attention distillation, and rolling learning techniques incorporated into our approach. Section 4 presents a detailed comparison of model performances and discusses the key results derived from the experiments. Finally, Section 5 provides a comprehensive summary of the study’s findings and contributions.

## 2. Data Processing in Injection Molding Experiments

### 2.1. Data Acquisition Platform

**Figure 2:**
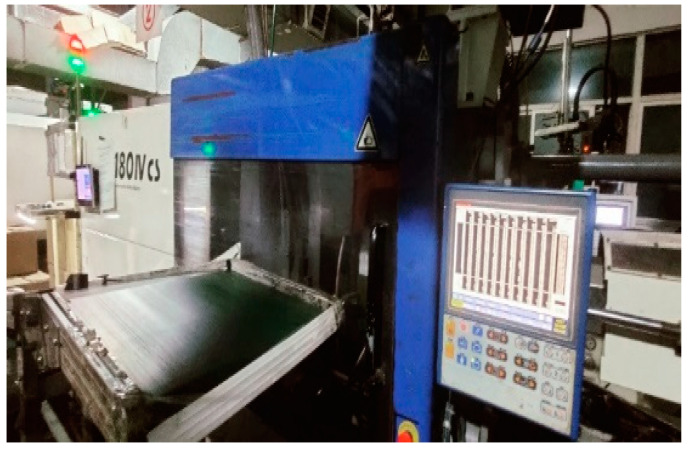
Injection machine Si-180IV_EHEXA284950-0505.

The injection molding machine utilized in this experiment is depicted in Figure 2 and is manufactured by Rui Xiang Plastics Co., Ltd. (Shantou, China). The identification number of the machine is Si-180IV_EHEXA284950-0505, and it is classified as a servo energy-saving drive model. This machine features an innovative V-type molding device and incorporates a newly designed shooting mechanism characterized by high rigidity and flexible stability.

**Figure 3:**
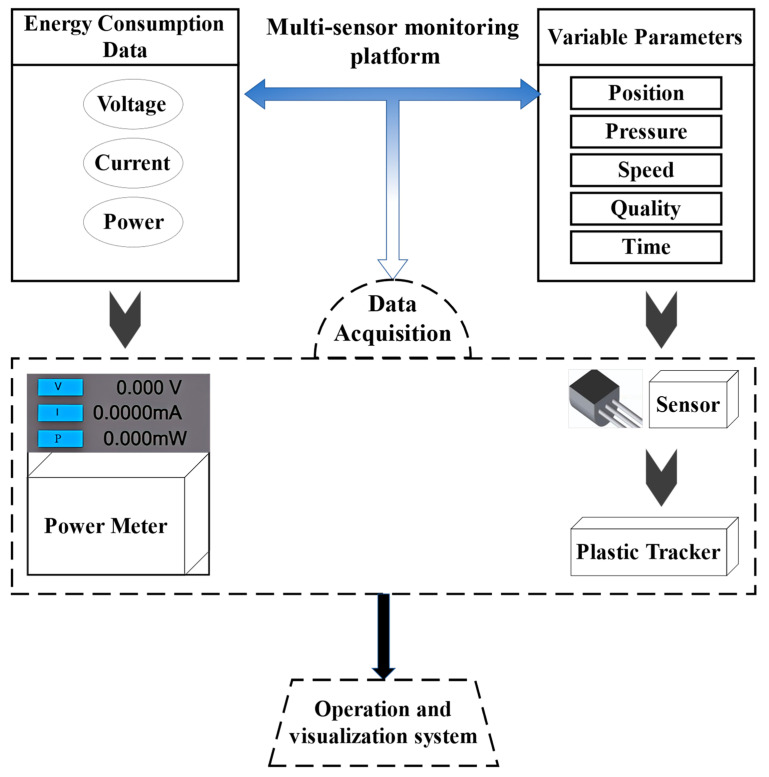
Schematic diagram of the multi-sensor platform.

The injection components processed in this study are handle inner buckle parts, measuring 82 mm in length, 45.9 mm in width, and 47.3 mm in height. The injection molding experiments utilize a blend of ABS and PBT in a mixing ratio of 70:30. The operational parameters of the injection molding process can be primarily categorized into temperature, speed, time, pressure, position, voltage, and current. In this study, using the data acquisition platform integrated into the machine, we collected real-time values for key parameters, including V-P switching position, remaining position, shrinkage time, mold opening time, and mold off time. Figure 3 provides a schematic diagram of the data measurement platform within the machine.

### 2.2. Data Processing Procedure

During the injection molding process, the generated data typically exhibit strong continuity. However, due to interference factors such as vibrations and temperature fluctuations inherent to the molding process [18,19], the sensor-collected data often suffer from missing values or extreme outliers, etc. To ensure data integrity and continuity, which are crucial for accurately analyzing and monitoring energy consumption, this study employs a fixed-range method to replace outliers. For instance, each component of the injection molding machine operates within a predefined range, and data exceeding these upper and lower bounds are replaced with the corresponding maximum or minimum values. In the case of power data exceeding the set limits, the outlier is substituted by the average of adjacent data points. For handling missing data, the KNN method is applied, which leverages the similarity between data points to fill in the missing values, with more accurate imputations when dealing with multiple missing values [20]. 

The main measured variables for the injection molding experiment are summarized in Table 1.

The relationships between various variables exhibit complexity, including linear and nonlinear characteristics, owing to the correlations of multiple influencing factors. To alleviate the computational burden of the model, enhance the operational efficiency, and effectively capture the correlations among variables, only those variables exhibiting strong effects on energy consumption are selected as model inputs. Linear relationships are evaluated using Pearson’s correlation coefficient [21], while nonlinear correlations are assessed through Spearman’s rank correlation coefficient [22]. In this context, a correlation coefficient closer to zero indicates a weaker association between variables.

**Figure 4:**
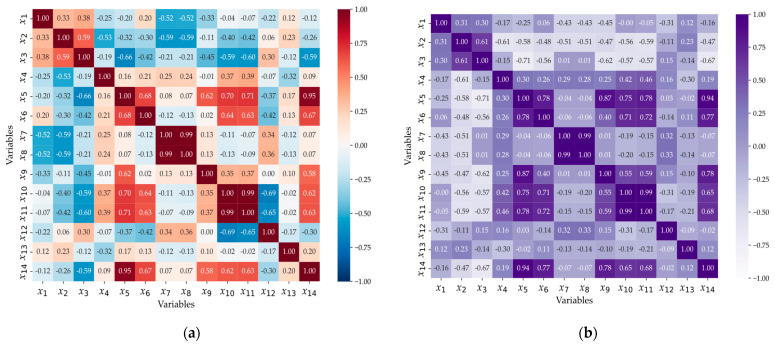
The coefficient heat map: (**a**) Spearman coefficient and (**b**) Pearson coefficient.

As illustrated in Figure 4, two pairs of variables, *x*_7_ and *x*_8_, as well as *x*_10_ and *x*_11_, exhibit (a) Spearman and (b) Pearson correlation coefficients as high as 0.99. These near-perfect correlations indicate a high degree of both linear and monotonic association between the variables, suggesting that they carry almost identical information. Such high correlation coefficients are indicative of potential multicollinearity, which can negatively impact the stability and interpretability of the model. To mitigate the risk of multicollinearity and redundancy, we selected one variable from each pair that demonstrated a stronger correlation with the target variable, *x*_14_, discarding the less correlated counterpart.

**Figure 5:**
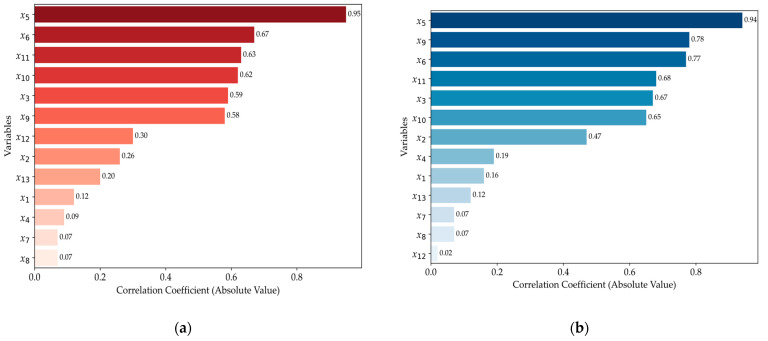
Correlation coefficient correlated with *x*_14_: (**a**) Spearman coefficient and (**b**) Pearson coefficient.

For example, Figure 4 highlights that variables *x*_3_, *x*_5_, *x*_6_, *x*_9_, *x*_10_, and *x*_11_ are significantly correlated with *x*_14_, either through (a) Spearman or (b) Pearson coefficients. Notably, *x*_11_ exhibits a marginally stronger correlation in both Pearson (0.68) and Spearman (0.63) analyses, leading to the decision to retain *x*_11_ and eliminate *x*_10_ to maintain model parsimony. Also, Figure 5b reveals that *x*_2_ presents a Pearson correlation coefficient of −0.47 (in Figure 5, the absolute values are shown), indicating a moderate negative linear correlation with *x*_14_, which suggests that, as *x*_2_ increases, *x*_14_ tends to decrease, albeit not in a perfectly linear manner. However, Figure 5a shows that the Spearman correlation coefficient for *x*_2_ is notably lower, at −0.26, indicating a weak monotonic relationship. This discrepancy between the two correlation measures suggests that, while *x*_2_ shares a linear relationship with *x*_14_, this relationship is not uniformly consistent across the entire data range, potentially due to the presence of local nonlinearities or the influence of outliers. Such behavior could indicate that *x*_2_ and *x*_14_’s relationship is sensitive to specific data points or particular regions of the data.

In summary, to ensure the robustness and interpretability of the model, we retained both highly and moderately correlated variables and selected *x*_2_, *x*_3_, *x*_5_, *x*_6_, *x*_9_, and *x*_11_ for further analysis. This selection strategy was designed to balance the inclusion of informative variables while mitigating multicollinearity and maintaining model efficiency.

## 3. Informer Model and Rolling Learning Prediction

The Transformer model, introduced in 2017 [23], has gained significant attention for its effectiveness in time sequence prediction within NLP. Unlike traditional RNN [24] and CNN [25], the Transformer employs a multi-head self-attention mechanism and feed-forward networks, allowing for efficient processing of long sequences through parallel computing [26]. This architecture enhances both training efficiency and task performance across various NLP applications. However, as sequence lengths increase, the Transformer exhibits limitations, including high memory consumption, inefficiencies in encoding/decoding, and increased time complexity, which restricts its application in large-scale temporal data prediction.

To address these challenges, the Informer model was introduced with several key improvements. By leveraging techniques such as model pruning, quantization, and structural innovations, the Informer significantly reduces computational and memory overhead while maintaining prediction accuracy, facilitating its deployment on embedded and edge devices [27]. Key enhancements include the sparse attention mechanism, self-attention distillation, and a generative decoder.

The sparse attention mechanism optimizes memory usage and reduces time complexity by focusing on essential key factors, improving efficiency in processing large datasets [28]. Self-attention distillation reduces input layers and parameters, effectively capturing long-term dependencies while enhancing generalization and minimizing input redundancy [29]. Finally, the generative decoder predicts entire long sequences in a single step, accelerating inference and improving efficiency for real-time applications. These innovations enable the Informer to handle long-sequence predictions more effectively, making it suitable for practical, large-scale temporal data tasks.

**Figure 6:**
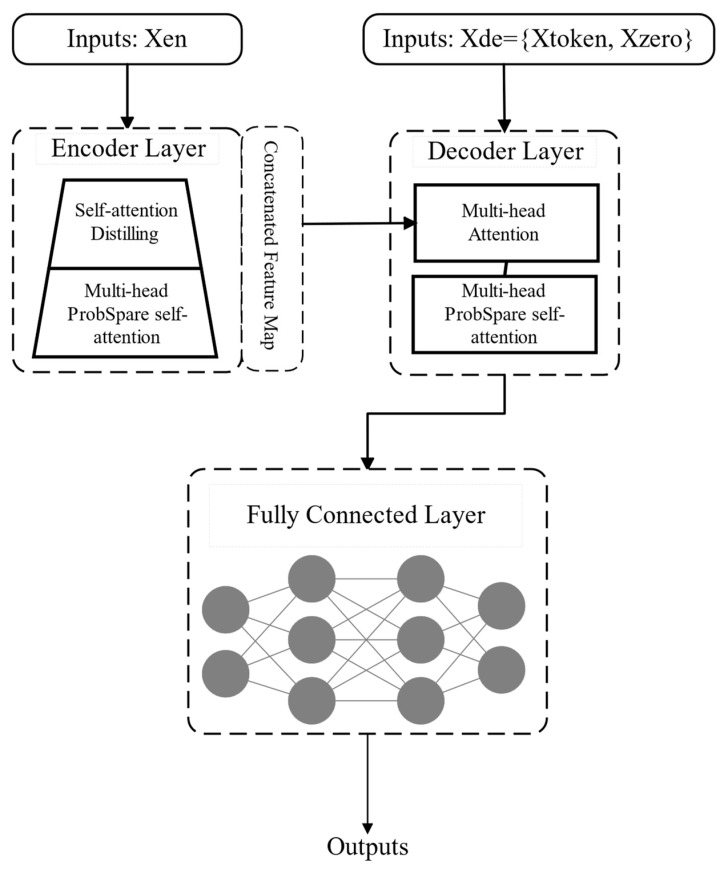
Structural diagram of the Informer model.

Figure 6 outlines the Informer model’s framework, with the encoder on the left and the decoder on the right. The Informer’s key improvement lies in its structural optimization of both components, enabling faster processing of long sequences. The encoder transforms the input sequence into hidden representations, leveraging sparse attention and self-attention distillation to capture critical information efficiently. Sparse attention enhances computational speed, while self-attention distillation refines feature extraction. The decoder then utilizes a generative mechanism to produce target sequence predictions by interacting with the encoder’s hidden representations through attention. This interaction allows the decoder to focus on relevant parts of the input, improving prediction accuracy and stability. Together, these mechanisms make the Informer highly effective for long-sequence predictions.

### 3.1. Encoder Module of the Informer Model

**Figure 7:**
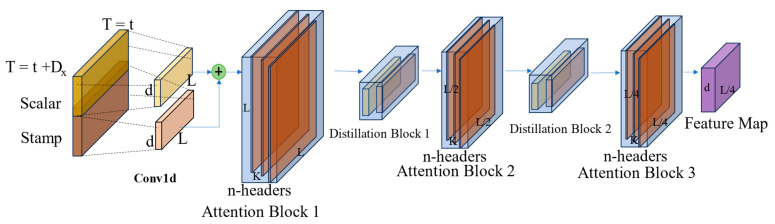
Encoder module of the Informer model.

Figure 7 is a single stack of the encoder structure in the Informer model, showing its complexity and the multi-level feature in detail. Combining the multi-head attention mechanism and the convolution operations with the maximum pooling operation and the self-attention distillation technique, the encoder can efficiently extract and integrate multilevel features of the time series data.

The primary role of the Informer model’s encoder is to transform input sequences into higher-level abstract representations [30]. Each encoder layer comprises a multi-head self-attention mechanism and a feed-forward neural network. The self-attention mechanism computes correlations across different positions within the sequence, allowing the model to prioritize critical time steps and features. Simultaneously, the feed-forward network applies nonlinear transformations to enhance feature extraction and the model’s representational capacity.

The core of the Informer consists of a stack of multiple encoder layers, where each layer extracts essential features and passes them to subsequent layers for deeper processing. The self-attention mechanism utilizes dot product matrices to compute positional correlations, enabling the model to focus on key time steps. The hidden representations at each step are processed and combined, eventually forming the encoder’s output.

A key innovation in the Informer model is its distillation operation. By applying hierarchical distillation to the encoder stack, the model simplifies its structure while improving generalization. This operation reduces the complexity of the encoder without sacrificing accuracy. The distillation operation can be represented by the following formula [17]:

(1) $$X_{j+1}^{t}=MaxPool\left(ELU\left(Conv1d\left({\left[{X_{j}}^{t}\right]}_{AB}\right)\right)\right)$$

where ${\left[X_{j}^{t}\right]}_{AB}$ represents the output of the multi-headed ProbSparse self-attention layer in the precious layer, and $X_{j+1}^{t}$ is the output of distillation in this layer; $Conv1d\left(\cdot \right)$ performs as a 1D convolutional filter;$ELU\left(\cdot \right)$ is used as the activation function; $MaxPool\left(\cdot \right)$ is finally applied to down-sample the processed data, retaining essential feature information while reducing data dimensionality. The distillation process enables efficient storage and processing without significant loss of critical information.

### 3.2. Decoder Module of the Informer Model

The decoder module is designed to generate the prediction of long sequences through a concise forward process. The model adopts the conventional decoder structure, which contains two identical multi-head attention layers. It solves the problem of high time complexity in long sequence data prediction by using a generative prediction method [31]. The input vector of the decoder is shown as follows:

(2) $$X_{\mathrm{feed}\_\mathrm{de}}^{t}=\mathrm{Concat}\left(X_{\mathrm{token}}^{t},X_{0}^{t}\right)\in {\mathbb{R}}^{(L_{\mathrm{token}}+L_{y})\times d_{\mathrm{model}}}$$

where $X_{token}^{t}$ is the start token; $L_{token}$ is the length of the long sequence selected from the input sequence and taken as the leading sequence that needs to be predicted. $X_{0}^{t}$ is a placeholder of the target sequence, and it should be zero. Through introducing the masked multi-head attention into the sparse self-attention mechanism, it will avoid the autoregressive phenomenon of $X_{0}^{t}$, because it will stop each position from noticing the next position. In the final output phase, the model obtains the prediction results through the fully connected layer, which is able to achieve the ability of univariate or multivariate prediction. The generative structure is integrated into the encoder so that the predicted sequence can be generated at once without gradual dynamic decoding. Owing to this, it significantly reduces the decoding time and improves the prediction efficiency.

### 3.3. Self-Attention Mechanism of the Informer Model

The self-attention mechanism was originally defined by the three matrix tuple inputs (Q, K, and V), which perform scaled dot product operations. The initial formulas are as follows [23]:

(3) $$A(Q,K,V)=\mathrm{Softmax}\left(\frac{QK^{T}}{\sqrt{d}}\right)V$$

where d is the input dimension and $Q\in R^{L_{Q}\times d}$, $K\in R^{L_{K}\times d}$, and $V\in R^{L_{V}\times d}$ represent query, key, and value, respectively. Both the key and the value are paired, and the query can get the corresponding value using the key. To further analyze the self-attention mechanism, the attention of the i-th query is defined as a kernel smoother in probabilistic form, which is defined as follows [17]:

(4) $$A(q_{i},K,V)=\sum_{j}\frac{k(q_{i},k_{j})}{\sum_{l}k(q_{i},k_{l})}v_{j}=E_{p(k_{j}|q_{i})}[V_{j}]$$

where $p\left(k_{j}|q_{i}\right)=\frac{k(q_{i},k_{j})}{\sum_{l}k(q_{i},k_{l})}$, $k(q_{i},k_{j})=\exp\left(\frac{q_{i}k_{j}^{T}}{\sqrt{d}}\right)$ are the asymmetric indices. This kind of self-attention mechanism calculates the probability $p\left(k_{j}|q_{i}\right)$ through the dot product operation, reaching the quadratic time complexity. Additionally, it needs the spatial complexity of $O\left(L_{Q}|L_{K}\right)$ while calculating.

By regulating self-attention through qualitative analysis, the probability distribution of the self-attention mechanism has the potential sparsity, showing a trend of long-tailed distribution. It means that a very small amount of dot products contributes the main attention weight, so the contribution of other dot products can be ignored. In that case, the contribution of the partial query to a value can be calculated without calculation. Consequently, the KL (Kullback–Leibler) divergence is introduced to compute and distinguish the important query. It is as follows [17]:

(5) $$KL(q∥p)=\ln L_{k}\sum_{j=1}^{L_{k}}e^{\frac{q_{i}k_{j}^{T}}{\sqrt{d}}}-\frac{1}{L_{k}}\sum_{j=1}^{L_{k}}\frac{q_{i}k_{j}^{T}}{\sqrt{d}}-\ln L_{k}$$

Excluding the constant, the sparsity formula of the i-th query is:

(6) $$M(q_{i},K)=\ln L_{k}\sum_{j=1}^{L_{k}}e^{\frac{q_{i}k_{j}^{T}}{\sqrt{d}}}-\frac{1}{L_{k}}\sum_{j=1}^{L_{k}}\frac{q_{i}k_{j}^{T}}{\sqrt{d}}$$

where the first item $q_{i}$ is the LogSumExp (LSE) on all keys. If the i-th query has a large value $M(q_{i},K)$, it means that the corresponding probability of attention distribution has greater diversity. This shows that, in the self-attention mechanism, the chance of including the dominant dot product pair in the head domain of the long-tailed self-attention distribution is high. Based on this analysis, by allowing each key to handle only u dominant query, the ProbSparse Self-attention formula is as follows [17]:

(7) $$A(Q,K,V)=\mathrm{Softmax}\left(\frac{\bar{Q}K^{T}}{\sqrt{d}}\right)V$$

In the ProbSparse Self-attention, a kind of sparse matrix of the same size as the query vector q is used. This sparse matrix contains only the Top-u query under the sparse evaluation; while u is controlled by a fixed sampling factor c, let $u=c\cdot \ln L_{Q}$ [32]. In this way, when ProbSparse Self-attention is in each matching query key, ProbSparse Self-attention only needs to perform u dot product operations, so that the memory overhead of each layer is reduced to $O(L_{K}\ln L_{Q})$. However, traversing all measured query $M(q_{i},K)$ requires computing each dot product pair, with potential numerical stability problems. Therefore, the max–mean metric is introduced as a new indicator for assessing the sparsity of the query.

(8) $$\bar{M}(q_{i},K)=\max_{j}\left(\frac{q_{i}k_{j}^{T}}{\sqrt{d}}\right)-\frac{1}{L_{K}}\sum_{j=1}^{L_{K}}\frac{q_{i}k_{j}^{T}}{\sqrt{d}}$$

In the case of the long-tail distribution, $U=L_{K}\ln L$ dots are randomly selected to calculate $\bar{M}(q_{i},K)$, and the other dot product pairs are filled to zero. The reason for choosing the number of Top-u to be the max operator in $\bar{Q}$ and $\bar{M}(q_{i},K)$ is that its sensitivity to zero is low and numerically stable. In practice, the input lengths of the query and key in self-attention computation are often equal, like $L_{Q}=L_{K}=L$. This reduces the temporal and spatial complexity of ProbSparse Self-attention to O(LlnL).

### 3.4. Rolling Learning Prediction

Rolling learning prediction enhances time series models by dynamically updating the training dataset, enabling models to capture evolving patterns and improve prediction accuracy. Liu et al. [33] applied this approach with LSTM for battery performance prediction, allowing continuous parameter updates for future state estimation. Similarly, Guo et al. [34] demonstrated that rolling learning improves MPC model accuracy, particularly in real-time energy management of plug-in hybrid electric vehicles. These studies underscore the potential of rolling learning for managing dynamic temporal data and complex nonlinear systems, achieving precise predictive control.

The original Informer model, though effective for fixed-length time series predictions, shows declining accuracy over time due to its limited adaptability to dynamic input data [35]. Rolling learning addresses this by continuously updating the model to capture new data trends. Traditional time series models rely on static historical data, limiting their adaptability in dynamic environments. In complex processes like injection molding, where fluctuating parameters impact energy consumption, static models struggle to capture real-time variations.

Rolling learning prediction, utilizing techniques such as sliding windows, addresses this challenge by dividing time series data into overlapping subsequences. The model is trained on each subsequence and updated continuously, enhancing the adaptability to new data and shifting trends. This approach improves prediction accuracy and enables the model to handle non-stationary and abrupt changes in the data [36].

By integrating rolling learning prediction into the Informer model, an improved RL-Informer model is proposed. The steps are as follows:

1. Use the original Informer model to predict future values for the next time periods and generate the initial prediction sequence.
2. Feed the predicted values back into the model to update the input sequences.
3. Use the updated input sequences to predict the next time periods, continuously refining the model with the latest date.

### 3.5. Evaluation Metric

The performance of each model was assessed using several evaluation metrics, including NRMSE, RMSE, MAE, and R^2^. NRMSE calculated by Equation (9) provides a normalized measure of the prediction error, allowing for a relative assessment of model accuracy across different scales [37]. RMSE calculated by Equation (10) is a standard metric in regression analysis that calculates the average magnitude of errors between predicted and actual energy consumption data [38]. MAE defined by Equation (11), on the other hand, measures the average absolute deviation, offering an intuitive sense of prediction accuracy [39]. The R^2^ metric defined by Equation (12) indicates the proportion of variance in the actual data that is explained by the model [40], reflecting its ability to capture the underlying patterns in energy consumption. Lower NRMSE, RMSE, and MAE values, combined with higher R^2^ values, signify greater accuracy and robustness in the model’s predictions. Equations for calculating NRMSE, RMSE, MAE, and R^2^ are given below:

(9) $$\mathrm{NRMSE}=\frac{\sum_{i=1}^{n}{\left({\hat{y}}_{i}-y_{i}\right)}^{2}}{\sum_{i=1}^{n}{y_{i}}^{2}}$$

(10) $$\mathrm{RMSE}=\sqrt{\frac{1}{n}\sum_{i=1}^{n}{\left(y_{i}-{\hat{y}}_{i}\right)}^{2}}$$

(11) $$\mathrm{MAE}=\frac{1}{n}\sum_{i=1}^{n}\left|y_{i}\right|$$

(12) $$R^{2}=1-\frac{\sum_{i=1}^{n}{\left(y_{i}-{\hat{y}}_{i}\right)}^{2}}{\sum_{i=1}^{n}{\left(y_{i}-\bar{Y}\right)}^{2}}$$

where $y_{1}$,$y_{2}$,…,$y_{n}$ represent the actual data; ${\hat{y}}_{1}$,${\hat{y}}_{2}$,…,${\hat{y}}_{n}$ are the predicted data; $\bar{Y}$ is the mean value of the actual data; n is the number of the samples.

## 4. Energy Consumption Prediction and Results Analysis

### 4.1. Process Structure

**Figure 8:**
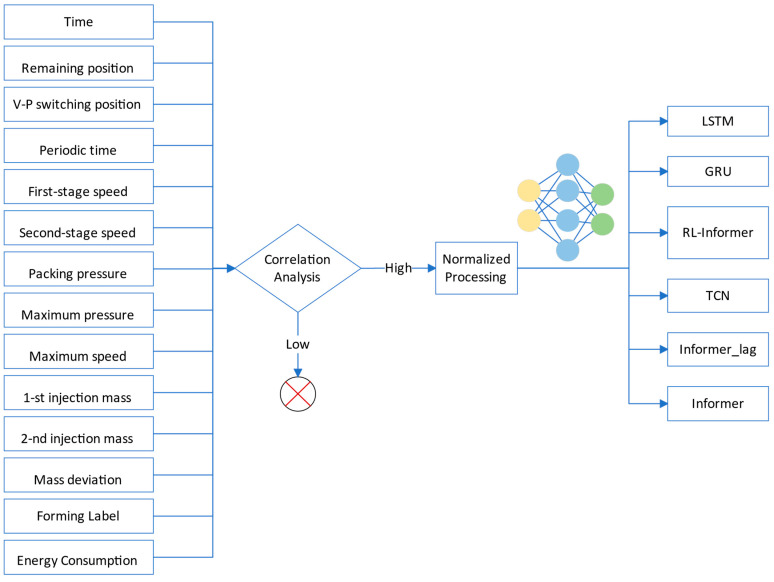
Structure of the energy consumption prediction.

In Section 2, six key variables influencing energy consumption in the injection molding process were identified. To eliminate the impact of varying magnitudes and dimensions among these variables, the max–min normalization technique [41] was applied, transforming the data into the [0, 1] range. This ensured that each variable contributed proportionally to the model without introducing biases due to differences in scale. Following normalization, other advanced models, including LSTM, GRU, and TCN, were implemented for comparison against the RL-Informer model, providing a comprehensive performance benchmark. The overall process structure is illustrated in Figure 8. Note that, in order to highlight the benefits of incorporating rolling learning prediction, the pure Informer model and its variant, Informer_lag, are also compared. They will be detailed later.

### 4.2. Performance Comparison

In this experiment, data from 180 injection molding cycles were collected and divided into training, validation, and testing sets in a 7:2:1 ratio. To establish a robust comparative framework for evaluating the performance of the Informer model in predicting energy consumption in injection molding, several advanced temporal prediction models were introduced, including LSTM, GRU, and TCN. These models are well suited for handling complex time series data and capturing long-term dependencies with nonlinear features. By comparing the prediction outcomes of these models, the accuracy and generalization capabilities of the Informer and RL-Informer models can be comprehensively assessed, thereby providing a strong basis for determining their superiority in this application.

The GRU is an efficient recurrent neural network designed for capturing long-term dependencies in time series data, offering a balance between accuracy and computational cost [42]. In contrast, the TCN uses dilated convolutions to model long-range dependencies, providing faster training and improved scalability for time series forecasting [43]. The prediction curves and corresponding error plots for the GRU and TCN models are shown below.

**Figure 9:**
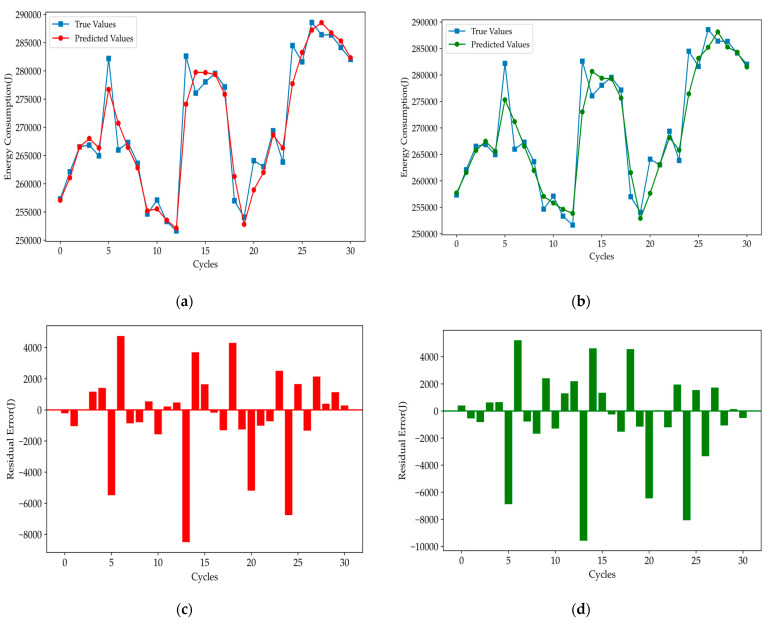
Prediction results for (**a**) GRU and (**b**) TCN, and residual errors for **(c**) GRU and (**d**) TCN.

Figure 9 shows that the GRU and TCN models capture short-term fluctuations well but struggle with larger residual errors in areas of rapid change. However, they can capture the overall change characteristics of the data more accurately. Then, we test the initial Informer model, and the predicting curve and error results are as follows. 

**Figure 10:**
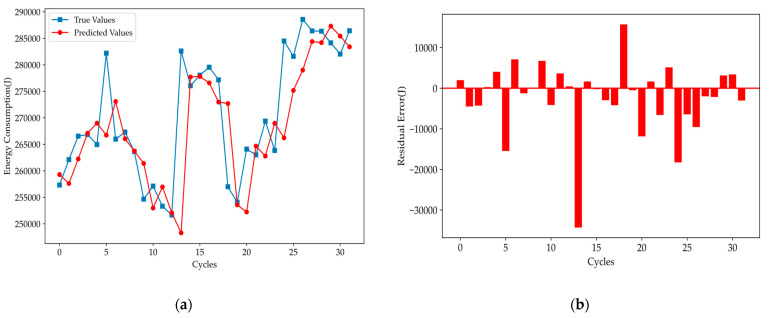
Prediction results: (**a**) comparison of true and predicted values, (**b**) residual error of the initial Informer model.

**Figure 11:**
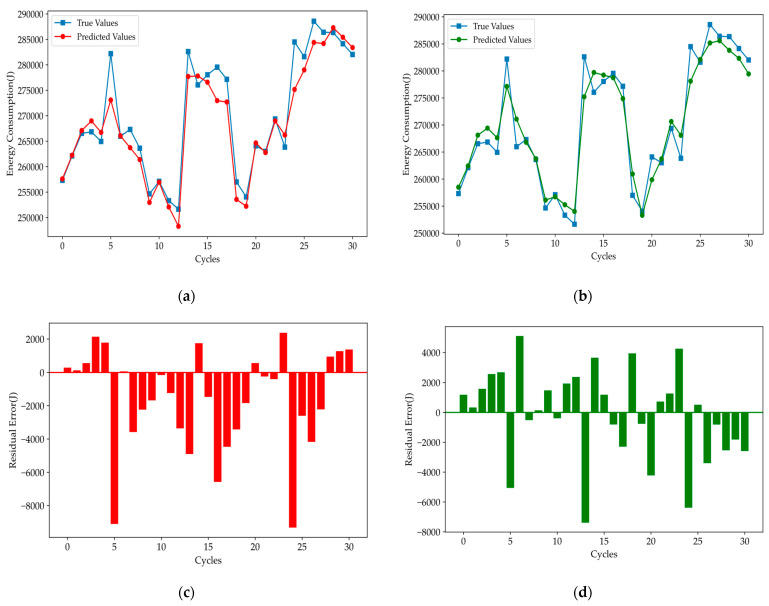
Prediction results for (**a**) Informer_lag and (**b**) LSTM, and residual error for (**c**) Informer_lag and (**d**) LSTM.

Figure 10 shows that, for the Informer model, the predicted values generally track the variation trends of the true values across most cycles. However, the predicted curve exhibits an obvious lag in output, which reduces the overall prediction accuracy. To address this, output lag processing was incorporated into the Informer model, resulting in the new variant, Informer-lag. Furthermore, a comparison with the LSTM model was conducted. The results are shown in Figure 11.

**Figure 12:**
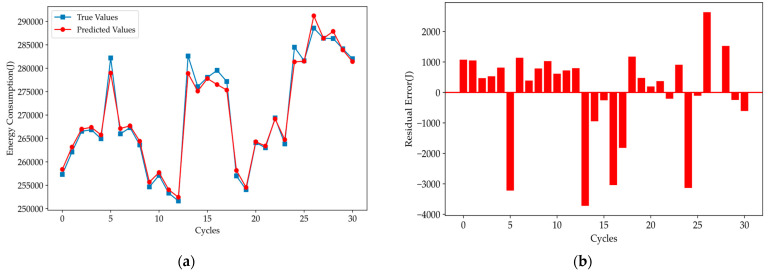
Prediction results: (**a**) comparison of true and predicted values, (**b**) residual error of the RL-Informer model.

Figure 11 shows that the predicted curve of Informer_lag closely follows the true values, with significant reductions in both extreme values and mean errors, indicating a notable improvement in the model performance. However, when compared to the prediction curve of the LSTM model, the Informer model with anti-lag treatment still showed slightly lower tracking accuracy. To address this, we applied a rolling learning prediction technique to enhance the original Informer model. The prediction results of the improved RL-Informer model are presented in Figure 12.

As shown in Figure 12, the prediction accuracy of the improved RL-Informer model has significantly increased. The model demonstrates precise tracking of the true energy consumption trends across all cycles, with a noticeable reduction in both the extreme value and mean errors. This improvement is further highlighted by the residual error plot, where the error magnitudes are substantially lower compared to previous models.

The RL-Informer model exhibits superior performance in capturing the complex dynamics of energy consumption, effectively reducing prediction discrepancies. This enhanced accuracy, particularly in the reduction of residual errors, underscoring the model’s robustness and its ability to generalize well across different segments of the time series. Compared to the GRU, TCN, and LSTM models, the RL-Informer model delivers more reliable forecasts with minimized prediction errors, making it a highly effective solution for time series energy consumption forecasting.

### 4.3. Performance Analysis

Table 2 presents the evaluation of different models in energy consumption prediction, with RL-Informer clearly outperforming all others. It achieves the lowest root mean square error (RMSE, 0.0758) and mean absolute error (MAE, 0.0562), demonstrating the smallest prediction error, and the highest coefficient of determination (R², 0.9831) not only demonstrates the model’s superiority in explaining the variance within the data but also indicates the potential of the RL-Informer model to deliver accurate predictions in dynamic and complex industrial applications. This is particularly significant for energy management and optimization, as accurate predictions can help identify potential energy-saving opportunities, thereby enhancing the overall production efficiency. Additionally, its normalized root mean square error (NRMSE, 13.01%) highlights its robustness, outperforming both traditional models and pure Informer-based models across all key metrics.

**Figure 13:**
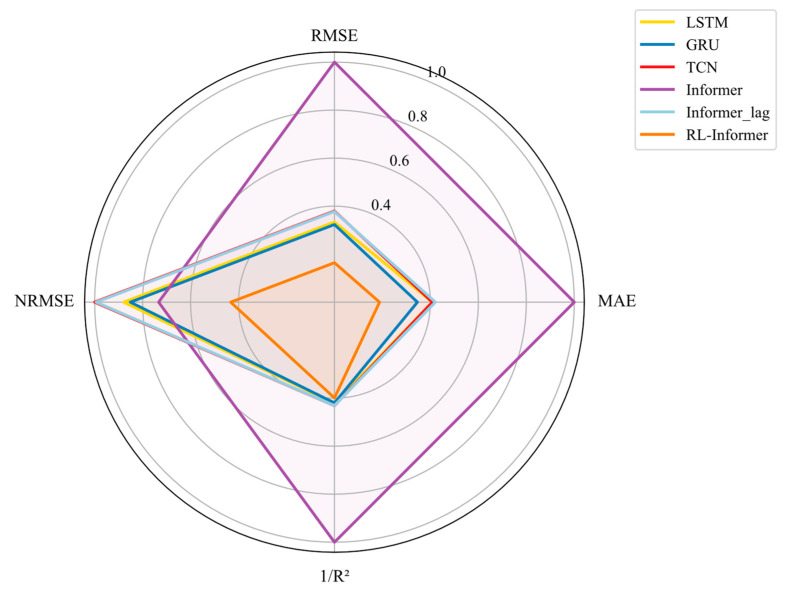
The model performance in the radar chart.

To facilitate a clearer comparison of the model performance, the evaluation metrics from Table 2 were normalized, with R^2^ replaced by 1/R^2^ to ensure consistency, whereby lower values indicate better performance in the models. The radar chart in Figure 13 visualizes these results, comparing six time series forecasting models—GRU, TCN, LSTM, Informer, Informer_lag, and RL-Informer—across four key metrics: RMSE, MAE, 1/R^2^, and NRMSE. The radar chart reveals each model’s strengths and weaknesses at a glance. The Informer model notably occupies the largest area on the radar chart, with the RMSE, MAE, and 1/R^2^ values close to the outer boundary, indicating significant errors in capturing the overall data trends and magnitudes. With the incorporation of lag-based adjustments, Informer_lag demonstrates considerable improvements in RMSE, MAE, and 1/R^2^, indicating enhanced data fitting capabilities and increased sensitivity to trend variations. Nevertheless, the higher NRMSE observed for Informer_lag suggests limitations in accurately capturing relative errors, revealing potential weaknesses in its robustness against data variability. Despite these adjustments, the overall accuracy of the lag-enhanced Informer remains comparable to that of the TCN model while still trailing slightly behind the GRU and LSTM models in terms of overall predictive accuracy.

Further improvements were achieved by implementing rolling learning techniques, which led to substantial reductions across all the evaluated metrics, underscoring the model’s enhanced accuracy and adaptability. This trend is especially evident in RL-Informer, which displays the smallest area on the radar chart, with all metrics converging closer to the center. These results underscore RL-Informer’s superior performance, highlighting its efficacy in minimizing prediction errors comprehensively.

## 5. Conclusions

This study introduces the RL-Informer model as a robust solution for accurately predicting energy consumption in the injection molding process. By enhancing the baseline Informer model with output lag adjustment and integrating a rolling prediction mechanism, the RL-Informer demonstrated significantly improved predictive accuracy. Compared to conventional temporal models such as GRU, TCN, and LSTM, the RL-Informer achieved notable reductions in RMSE (0.0758), MAE (0.0562), and NRMSE (0.1301), alongside an R² increase to 0.9831. These results underscore the RL-Informer model’s exceptional adaptability and precision, establishing it as a highly effective tool for real-time energy consumption forecasting in dynamic industrial settings.

The results confirm the reliability and accuracy of the RL-Informer model, making it a robust tool for energy consumption prediction in the injection molding process. The integration of rolling prediction has proven effective in optimizing the model’s performance, providing a practical and reliable approach for energy monitoring and prediction in industrial applications.

Although the RL-Informer model demonstrated a strong performance in energy consumption prediction for injection molding processes, the dataset used was relatively small and focused on a specific machine type, which may limit the model’s generalizability to other contexts. Additionally, while correlation-based variable selection reduced the computational load, it may have overlooked complex nonlinear interactions among the variables. Future work could focus on expanding the dataset and applying more advanced variable selection techniques to further enhance the model prediction accuracy and applicability across different production environments. Moreover, the current study focuses on predicting energy consumption as a single target variable. In real-world industrial processes, multiple variables such as product quality, cycle time, and machine health play critical roles in the overall performance. Future research could extend the RL-Informer model to predict multiple variables simultaneously, allowing for a more comprehensive optimization of the production process. Multi-output prediction models, including multi-task learning approaches, should be explored to simultaneously forecast energy consumption alongside other key metrics. By doing so, researchers can gain deeper insights into how various factors interact and impact the efficiency and effectiveness of the injection molding process.

## Figures and Tables

**Table 1:** Data variables of injection molding experiments.

Variable	Meaning	Unit
*x*_1_	Time	s
*x*_2_	Remaining position	mm
*x*_3_	V-P switching position	mm
*x*_4_	Periodic time	s
*x*_5_	First-stage speed	mm/s
*x*_6_	Second-stage speed	mm/s
*x*_7_	Packing pressure	MPa
*x*_8_	Maximum pressure	MPa
*x*_9_	Maximum speed	mm/s
*x*_10_	1-st injection mass	g
*x*_11_	2-nd injection mass	g
*x*_12_	Mass deviation	g
*x*_13_	Forming label	/
*x*_14_	Energy consumption	J

**Table 2:** Prediction index of the timing prediction model.

Model	RMSE	MAE	R^2^	NRMSE
GRU	0.1492	0.1034	0.9345	25.59%
TCN	0.1752	0.1220	0.9096	30.06%
LSTM	0.1540	0.1219	0.9302	26.42%
Informer	0.4614	0.2988	0.3923	22.04%
Informer_lag	0.1741	0.1258	0.9108	29.87%
RL-Informer	0.0758	0.0562	0.9831	13.01%

## Data Availability

Data will be made available on request.

## Abbreviations

The following abbreviations are used in this manuscript:

RL-Informer	Rolling Learning Informer
NRMSE	Normalized Root Mean Square Error
RMSE	Root Mean Square Error
MAE	Mean Absolute Error
R^2^	coefficient of determination
GRU	Gated Recurrent Unit
TCN	Temporal Convolutional Networks
LSTM	Long Short-Term Memory
ABS	Acrylonitrile-Butadiene-Styrene co-polymers
PBT	Polybutylene terephthalate
KNN	K-nearest neighbor
NLP	Natural Language Processing
RNN	Recurrent Neural Networks
CNN	Convolutional Neural Networks
TFT	Temporal Fusion Transformer
MPC	Model Predictive Control
